# Design, Development, and Prototype Evaluation of a Mobile Application for Postoperative Care Following Breast Surgeries

**DOI:** 10.1155/ijta/3079287

**Published:** 2026-11-15

**Authors:** Mohammadreza Azangou-Khyavy, Sadaf Alipour, Arash Roshanpoor, Ramesh Omranipour, Mahsa Tavakol, Maryam Sarkardeh, Shiller HesamiAzar, Bita Eslami

**Affiliations:** ^1^ Breast Diseases Research Center (BDRC) Cancer Institute Tehran University of Medical Sciences Tehran Iran tums.ac.ir; ^2^ School of Medicine Shahid Beheshti University of Medical Sciences Tehran Iran sbmu.ac.ir; ^3^ Department of Surgery Arash Women′s Hospital School of Medicine Tehran University of Medical Sciences Tehran Iran tums.ac.ir; ^4^ Department of Computer YI.C. Islamic Azad University Tehran Iran tiau.ac.ir; ^5^ Department of Surgical Oncology Cancer Institute School of Medicine Tehran University of Medical Sciences Tehran Iran tums.ac.ir; ^6^ Department of Surgery Imam Khomeini Hospital Complex Tehran University of Medical Sciences Tehran Iran tums.ac.ir

**Keywords:** breast, electronic health, mHealth, postoperative, surgery, telemedicine

## Abstract

**Background:**

There is a lack of comprehensive, evidence‐informed mobile health (mHealth) applications specifically designed to address the postoperative self‐care needs of patients undergoing various types of breast surgeries. This study is aimed at designing and evaluating a mobile application for this purpose.

**Materials and Methods:**

This descriptive‐developmental study was conducted in three phases. In the first phase, the general elements and functional features of the application were identified based on patients′ informational needs using qualitative and quantitative approaches, with input from 10 clinical experts. In the second phase, the software was designed and developed as an Android‐based mobile platform, using Visual Studio 2022 with Xamarin. In the third phase, the application was evaluated through expert review involved in the application design process, and a questionnaire administered to 10 participants who used the application following breast surgeries.

**Results:**

Based on expert consensus, breast surgeries were classified into five main categories (partial breast surgeries [lumpectomy], mastectomies, breast reconstruction, esthetic surgeries, and axillary surgery) and their relevant subgroups. Data elements with a content validity ratio (CVR) greater than 0.62 retained. Following development of the primary prototype, the application content was approved by all clinical experts after minor revisions. Evaluation of internal consistency demonstrated excellent reliability of the questionnaire (Cronbach^′^s alpha = 0.97). Overall, participants reported high levels of satisfaction with different sections of the application.

**Conclusion:**

The developed mobile application appears to be a feasible and useful tool for postoperative self‐care management following breast surgeries. It may improve patient engagement and support postoperative recovery while potentially reducing complications. Future studies are required to evaluate its long‐term effectiveness and the integration of additional supportive features.

## 1. Introduction

Breast cancer is the most common cancer among women, with increasing incidence and mortality worldwide. Surgery remains the main treatment, usually combined with adjuvant therapies. Proper postoperative care is essential for improving recovery and reducing challenges faced by patients and their caregivers. Following the SARS‐CoV‐2 pandemic, the importance of home‐based self‐management and remote healthcare support became more evident, particularly for patients with special healthcare needs.

Self‐management is defined as an individual′s ability to manage symptoms, treatment‐related issues, lifestyle changes, and psychosocial impacts with support from healthcare professionals, family, and community [1]. Studies have shown that self‐management interventions can improve quality of life, reduce stress, and support self‐care among women with breast cancer [2].

In recent years, mobile health (mHealth) technologies have gained increasing attention, especially in developing countries and remote areas with limited healthcare access [3]. mHealth refers to medical or public health practices supported by mobile devices, including patient education, communication, monitoring, and treatment adherence [4, 5]. Mobile applications provide patients with easy access to health information regardless of time or location.

Previous studies have demonstrated several benefits of mHealth programs for breast cancer patients, including increased awareness, improved physical activity, reduced anxiety, and better emotional well‐being and quality of life [2]. Furthermore, unmet needs related to rehabilitation support, physiotherapy access, and reliable postoperative information after breast surgery may be addressed through evidence‐based mobile application [6].

Despite these advantages, there is currently no comprehensive Persian language self‐management application specially designed for women after breast surgery; whether for cancer treatment or cosmetic purposes. Existing online information is often scattered, untranslated accurately, or lacks scientific validity. Besides, the contents of the existing programs lack scientific support and are therefore not reliable. In addition, cultural differences, dietary habits, personal beliefs, and support systems vary across countries and should be considered in patient education tools.

To address this gap, our multidisciplinary research team developed a mobile‐based, postbreast surgery care application providing evidence‐based guidance for patients. This application was subsequently evaluated by both specialist physicians and postoperative patients.

## 2. Materials and Methods

### 2.1. Study Design

This descriptive‐developmental study is aimed at designing, developing, and evaluating a mobile application for postoperative self‐management and educational support following different types of breast surgery. The study was approved by the Ethics Committee of Tehran University of Medical Sciences (No: IR.TUMS.IKHC.REC.1400.330), and conducted in accordance with the Declaration of Helsinki. Written informed consent was obtained from all participants before enrollment. This study was performed in four phases: needs assessment and definition of application elements, design and development, application evaluation, and controlled distribution.

#### 2.1.1. Phase I: Definition of Elements and Need Assessment

The first phase aimed to identify the essential components of the application based on the informational and supportive needs of women who have undergone various breast surgeries. An electronic literature review was conducted using major databases and relevant websites with keywords including breast cancer, surgery, application, mobile app, software application, mHealth, electronic health, e‐health, telemedicine, and self‐management.

To define key application features, surveys and semi‐structured interviews were conducted with breast surgery patients and clinical experts. Based on these findings, a researcher‐developed questionnaire was designed and evaluated by 10 multidisciplinary specialists, including breast and cancer surgeons, nutritionists, physiotherapists, psychiatrists, technology experts, and dedicated researchers.

Content validity was assessed using both qualitative and quantitative methods. Experts reviewed items for clarity and relevance. The content validity ratio (CVR) was calculated using Lawshe′s criteria [7] with a minimum acceptable value of 0.62 for a 10‐member expert panel. Content validity index (CVI) was also assessed using item‐level relevance scoring, and items with I − CVI ≥ 0.78 were retained [8]. Scale‐level CVR and CVI were calculated as mean item scores.

#### 2.1.2. Phase II: Application Design and Development

Based on the identified requirements, the application prototype was designed collaboratively by clinicians, researchers, and software developers. Multiple meetings were conducted to align technical implementation with clinical needs.

The application was developed using Visual Studio 2022 with Xamarin.Forms, allowing efficient cross‐platform development using shared code architecture. XAMAL was used for user interface design, while C# was employed for application logic and database interactions. SQLite.NET was incorporated to create and manage the local database.

Xamarin was selected because of its cross‐platform capability and development accessibility at the time of implementation. Future versions are planned to migrate to .NET MAUI for long‐term maintainability and compatibility. The prototype was tested on Android Versions 11–15 across multiple devices with different screen sizes and hardware configurations, demonstrating stable performance.

The user interface and visual identity, including section‐specific logos, were designed by experienced graphic designers. During prototype refinement, feedback from clinical collaborators and application design experts was incorporated to improve usability and functionality. The app was tested on multiple Android smartphones with different screen sizes and hardware specifications.

Since this was an early prototype, no patient‐specific or sensitive health data were stored in the application. Instead of real‐time clinical monitoring or alerts, educational content highlighted red‐flag signs and instructed users to seek urgent medical consultation when necessary.

#### 2.1.3. Phase III: Application Evaluation

After development of the final prototype, the application was introduced to patients who had undergone breast surgery. A user guide was provided to facilitate application use. Usability and acceptability were assessed using a 20‐item Likert‐scale questions. The questionnaire evaluated four domains (Table 1): user friendliness (six items), ease of use (seven items), educational and motivational impact (six items), and overall satisfaction (1 item). Open‐ended questions were also included to collect qualitative feedback and suggestions. Questionnaire reliability was evaluated using Cronbach′s alpha coefficient. In addition, corrected item‐total correlations were examined to evaluate the contribution of each item to the overall scale reliability. Descriptive statistics, including mean and standard deviation (SD), were calculated for each item. A Cronbach′s alpha value of 0.70 or higher is considered acceptable for internal consistency.

**Table 1:** A questionnaire used for the evaluation of the application and user satisfaction.

Question	Strongly agree	Agree	Neutral	Disagree	Strongly disagree
User‐friendly					
1. The software has an attractive appearance.					
2. The information within the software is organized appropriately.					
3. The graphic elements, buttons, and colors used are suitable.					
4. The page design is clear and intuitive.					
5. The fonts used are easy to read.					
6. Learning is facilitated through the use of images.					
Ease of use					
7. The software is easy to use.					
8. Navigating to the next page or returning to the previous page is simple.					
9. Clear and necessary instructions are provided.					
10. Entering information is straightforward.					
11. Switching between pages is easy.					
12. Accessing information is convenient.					
13. The text explanations are easy to read and understand.					
Motivational and educational effects					
14. I am willing to use this software.					
15. I would recommend this software to others who have undergone surgery.					
16. The software effectively meets the informational needs of patients regarding proper self‐care methods post‐surgery.					
17. This software enhances the learning experience, making it easier and more effective.					
18. The information provided by the software is clear and comprehensible.					
19. The content of the software is excellent.					
Overall satisfaction					
	Very satisfied	Satisfied	Neutral	Dissatisfied	Very dissatisfied
20. Overall, satisfaction with the software.					

#### 2.1.4. Phase IV: Distribution and Installation

The application was initially distributed only within a controlled clinical environment and was not publicly released through app stores before validation. Access was restricted to patients referred by clinicians at affiliated research centers. Public release to the standard repositories was planned after final evaluation.

The prototype was developed specifically for Android devices because of their wider accessibility and use in the target population, facilitating rapid deployment and usability testing.

No personally identifiable information was collected or stored. Data were maintained locally on users′ devices using SQLite offline storage, and security depended on device‐level authentication. If a device was lost, the application could be reinstalled without affecting functionality, as no user‐specific data were retained.

The application was designed exclusively as an educational and self‐management support tool and was not intended to replace emergency medical care. Patients were instructed to contact their physicians immediately if warning symptoms occurred.

## 3. Results

### 3.1. Phase I

Initially, the software was planned to focus exclusively on breast cancer‐related surgeries. However, after consultation with clinical specialists, it was decided to expand the scope of the application to include all common breast surgeries. Based on expert consensus, breast surgeries were categorized into five main groups: partial breast surgeries (lumpectomy), mastectomies, breast reconstruction, esthetic surgeries, and axillary surgery. Breast reconstruction procedures were further divided into three subgroups: reconstruction using prostheses, reconstruction using distant flaps, and combined procedures involving both prostheses and flaps. Likewise, esthetic surgeries were classified into three subgroups: implant‐based procedure, mastopexies, and breast reduction surgeries. Surgeries performed simultaneously with axillary or other related procedures were also appropriately linked within the application structure (Figure 1).

**Figure 1:**
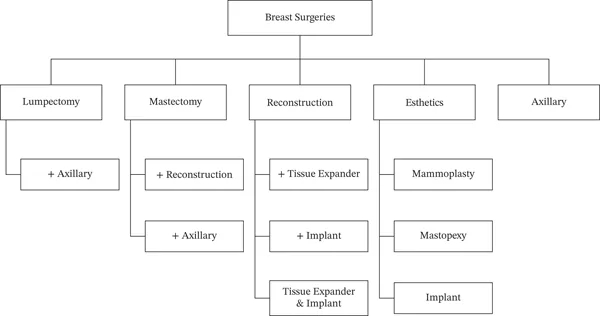
Breast surgeries included in the application.

According to Lawshe′s criteria, items with a CVR less than 0.62, such as marital status, employment, place of residence, time of disease diagnosis, offline consultation, and response to questions, were removed from the questionnaire (Table 2). In addition, medication time reminders, follow‐up visit reminders and other alert‐based features were removed because of substantial variability among patients, which limited the ability to generalized the content. S‐CVI for the entire questionnaire was 0.98, indicating excellent content validity. In the exercise section, videos were removed because of their large file size and potential download limitations. Instead, clear written instructions accompanied by images were provided. Table 3 presents the data elements of the application and their content format. Figure 2 illustrates selected application logos developed during the design process.

**Table 2:** Decisions on the various content items according to the I‐CVR and I‐CVI.

Item no.	Item statement	I‐CVR	I‐CVI	Decision
Identity information of individuals				
1	Name and family name	−0.20	0.40	Remove
2	Age	1	1	Retained
3	Education	0.80	0.90	Retained
4	Employment	0.20	0.60	Remove
5	Marital status	0.20	0.60	Remove
6	Place of residence	0.20	0.60	Remove
7	Time of disease diagnosis	0.60	0.70	Remove
8	Time of surgery	0.80	0.90	Retained
Information about the surgical service location				
9	Name of hospital	0.20	0.60	Remove
10	Name of surgeon	0.20	0.60	Remove
Surgery related data				
11	Type of surgery	1	1	Retained
12	Therapeutic surgeries such as lumpectomy, mastectomy, and axillary surgery	1	1	Retained
13	Reconstruction	1	1	Retained
14	Esthetics	0.80	1	Retained
15	General care	1	1	Retained
16	Wound and dressing care	1	1	Retained
17	Care of drains	1	1	Retained
18	Bra usage	1	1	Retained
19	Bathing	1	1	Retained
20	Nutrition, smoking, and alcohol consumption	0.80	1	Retained
21	Stress management skills	0.80	1	Retained
22	Massage	0.80	0.80	Retained
23	Complications and specific care	1	1	Retained
24	Breathing technique, physical activity, and exercise	1	1	Retained
25	Lymphedema	1	1	Retained
26	Medication time reminders	−0.60	0.30	Remove
27	Reminders for follow‐up visits	−0.60	0.20	Remove
28	Response to questions	−1	0	Remove
29	Offline consultation	−1	0	Remove
Patient feedback				
30	Survey questionnaire	0.80	1	Retained

*Note:* All items were calculated according to the 10 experts′ judgment. S‐CVR: 0.93, S‐CVI: 0.98. S‐CVR and S‐CVI are calculated using average accepted item‐levels.

**Table 3:** Educational content, its format, and logos in the application.

Title	Format	Logo
General care after surgery	Text	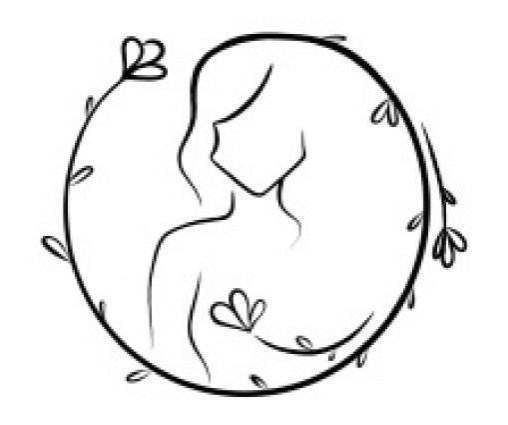
Wound and dressing care	Text	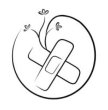
Care of drains	Text, video, electronic notebook	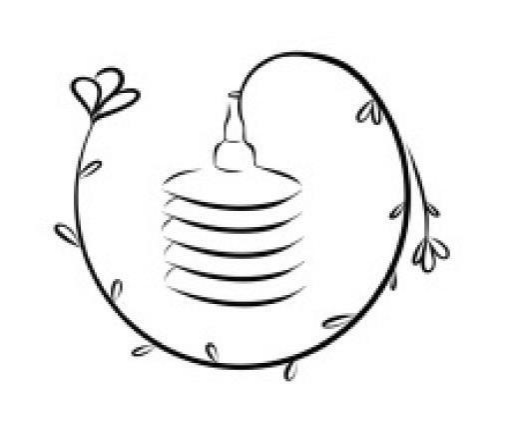
Bra usage after surgery	Text	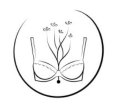
Bathing after surgery	Text	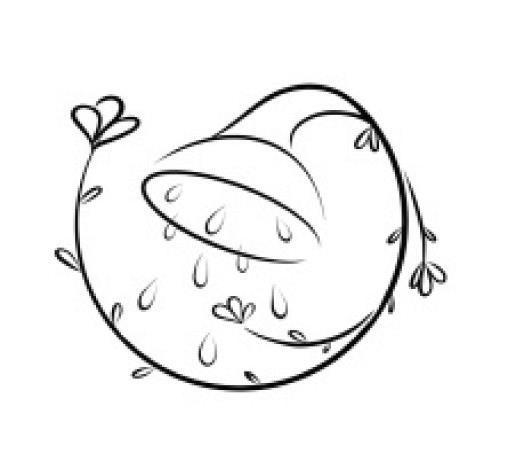
Nutrition, smoking, and alcohol consumption	Text	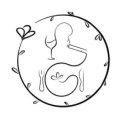
Stress management skills	Text, music	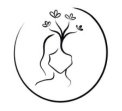
Massage	Text	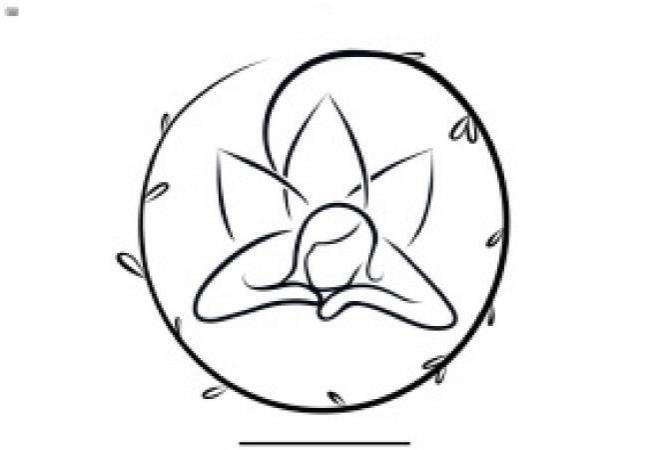
Complications and specific care	Text	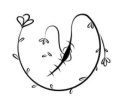
Breathing technique, physical activity, and exercise after surgery	Text, image	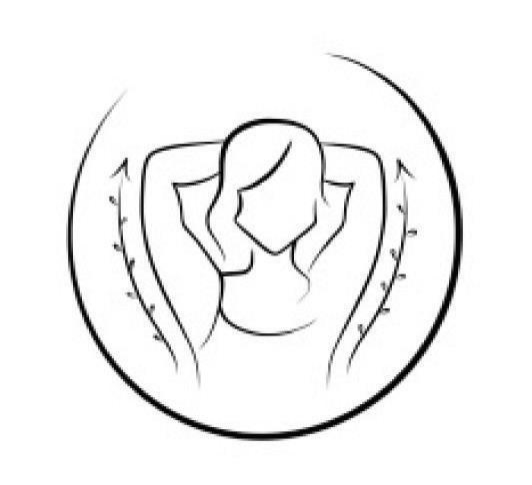
Lymphedema	Text, image	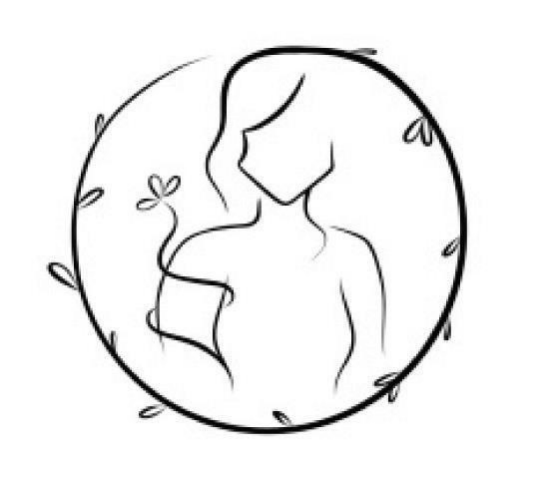
Survey questionnaire	Text	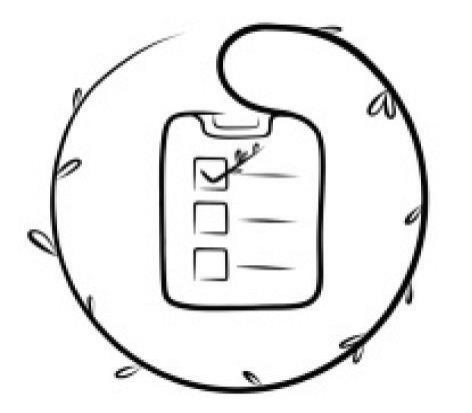

**Figure 2:**
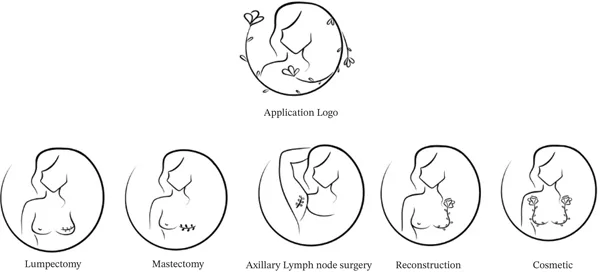
Designed logos for different surgeries.

### 3.2. Phase II

The final interface was developed according to user needs and included the following sections:

1. Welcome page: To emphasize that this mobile application is not intended to replace physician visits and is designed solely to support self‐care management, this page appears each time the user opens the application. Users must acknowledge this statement before signing in.
2. Type of surgery page: Since the application content was tailored according to the type of surgery, users were required to select the surgery they had undergone (Figure 3A).
3. Home page: This page displayed a list of educational materials based on the selected type of surgery. If a user made an incorrect selection, they could return to the previous page and modify their choice. Additionally, the “About Us” section was placed in the application logo area, providing information about all contributors involved in the development of the application (Figure 3B).
4. Educational content: The selected educational material was presented on this page. The content included text, images, videos, or a combination of these formats (Figure 3C). On certain pages, such as drain care, users were able to record their daily drainage volume in an electronic notebook while simultaneously receiving the necessary explanations.
5. Questionnaire: At the end of the educational content section, a link to an online survey questionnaire was provided to collect users′ feedback regarding the application.

**Figure 3:**
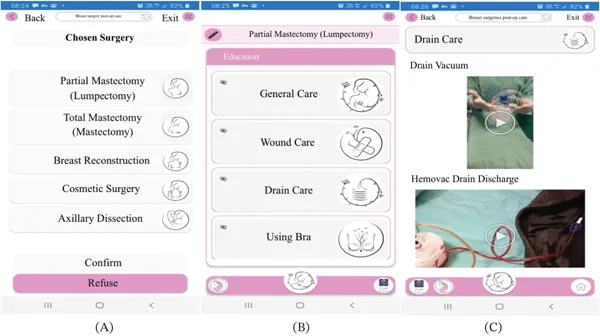
Surgical application interfaces. (A) Interface for selecting the type of surgical procedure, (B) educational content page for patient guidance, and (C) instructional page for postoperative drain care.

### 3.3. Phase III

During the final in‐person and online meeting, attended by the principal investigator, five clinical experts, and two individuals involved in the application design process, all components of the application were reviewed and tested. Only minor modifications were recommended and subsequently implemented, including correction of typographical errors, adjustment of color and fonts in specific sections, and converting the date format in the electronic notebook from the Gregorian calendar to the Solar Hijri calendar. The application was approved by all clinical experts, resulting in a 100% approval rate.

Subsequently, the application was made available to 10 postoperative patients for usability evaluation. The group included two patients who had undergone mastectomies with reconstruction, four patients who had undergone mastectomies with or without axillary surgery, and four patients who had undergone breast‐conserving surgery with or without axillary dissection. Participants ranged age from 36 to 58 years, and all had undergone surgery at Imam Khomeini Hospital, Tehran, Iran. Evaluation of internal consistency demonstrated excellent internal consistency (Cronbach^′^s alpha = 0.97). Cronbach′s alpha coefficients for the three subscales (user‐friendly, easy to use, and motivational and educational effects) were 0.90, 0.96, and 0.92, respectively, indicating excellent reliability. Item descriptive statistics and corrected item‐total correlations were shown in Table 4. The overall patient satisfaction rate was 100%, with all respondents reporting that they were either very satisfied or satisfied with the application. One patient suggested including additional information about sensitivity to sutures in the comments section. Two patients reported being very satisfied with the drain care section, particularly the video‐based demonstration.

**Table 4:** Item descriptive statistics and corrected item‐total correlations.

Item	Mean	SD	Corrected item–total correlations
Q1	4.4	0.5	0.72
Q2	4.6	0.5	0.89
Q3	4.6	0.5	0.89
Q4	4.6	0.5	0.48
Q5	4.8	0.4	0.81
Q6	4.6	0.5	0.89
Q7	4.8	0.4	0.81
Q8	4.6	0.5	0.81
Q9	4.8	0.4	0.81
Q10	4.8	0.4	0.89
Q11	4.8	0.4	0.81
Q12	4.6	0.5	0.89
Q13	4.8	0.4	0.81
Q14	4.6	0.5	0.89
Q15	4.5	0.5	0.26
Q16	4.5	0.5	0.86
Q17	4.5	0.5	0.86
Q18	4.5	0.5	0.86
Q19	4.5	0.5	0.86

Abbreviations: Q, question; SD, standard deviation.

No adverse events or safety concerns were observed during the testing phase. Since the study was prototype‐based and noninterventional, it did not involve treatment modification, clinical decision‐making or exposure to additional patient risk. Although the study did not strictly adhere to the mERA and CONSORT‐EHEALTH reporting frameworks, relevant principles of transparency and ethical reporting were considered throughout the development and evaluation process [9]. The study plan flowchart, including the outcomes and timeline for each phase, is presented in Figure 4.

**Figure 4:**
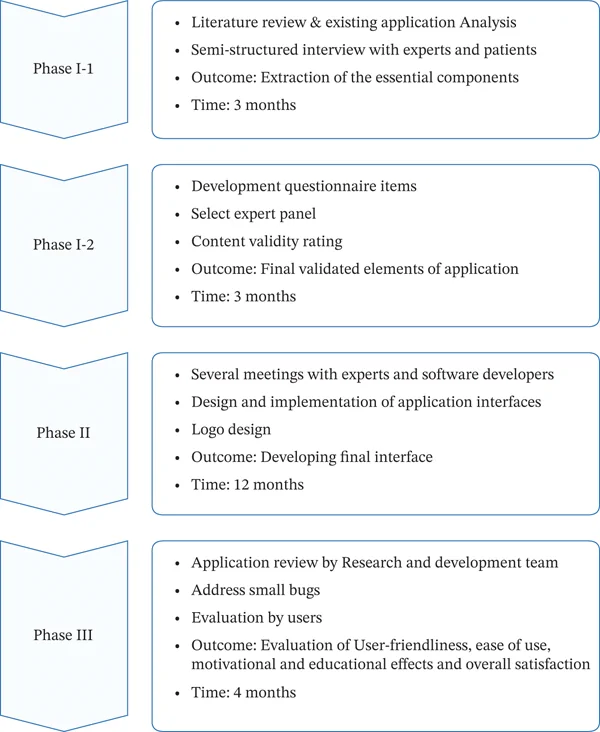
Study plan flowchart: outcomes and timeline for each phase.

## 4. Discussion

In the present study, a mobile application was successfully developed and tested among breast surgery patients. The application addresses multiple aspects of postoperative care and provides a comprehensive, accessible, and user‐friendly guide for patients. All participants who used the application reported it to be easy to use. Moreover, the application may support healthcare providers by reducing the amount of repetitive postoperative education required at hospital discharge and minimizing the risk of omission of important instructions.

Mobile applications in the field of breast cancer are generally categorized into two main groups: those developed for healthcare professionals and those designed for patients [10, 11]. Most existing breast cancer‐related mobile applications primarily focus on chemotherapy‐related symptoms management and its impact on patients′ quality of life [12–15]. Other studies have focused on mental health support and self‐assessment tools for early breast cancer detection [15, 16].

Despite the growing interest and demand for mHealth applications among breast cancer patients [6], comparatively limited attention has been given to postoperative care and surgery‐related symptom management. For example, an application developed in Singapore was mainly limited to monitoring wound and drain discharges after breast surgery [17]. In Iran, two mobile applications have been reported for breast cancer patients; however, both primarily focus on self‐care management of lymphedema [10, 18].

One of the major challenges in postoperative breast surgery care is the large number of patient concerns and questions have regarding optimal self‐care after discharge. Patients frequently express concerns about physical changes, symptoms, or complications that may arise following surgery. Providing comprehensive postoperative instructions can be time‐consuming for surgeons, and essential points may occasionally be overlooked, particularly in busy public hospital settings. This application was designed to address these challenges by providing accessible and structured postoperative self‐care information.

Furthermore, an essential aspect of postoperative care after axillary surgery is the prevention of lymphedema. In addition to guiding patients through the required exercise, the application provides important information regarding warning signs and necessary precautions to reduce likelihood of lymphedema development.

To the best of our knowledge, this is the first Persian‐language mobile application focusing on patient care after breast surgery. It addresses multiple aspects of self‐management ranging from surgery‐specific postoperative care recommendations to general postoperative care instructions. The application also covers many patients′ needs that have previously been identified in breast cancer care mobile applications [19]. Moreover, the growing use of mobile applications and social media among the Iranian population may facilitate better acceptance, accessibility, and adherence to this type of digital health intervention [20]. However, future studies are required to evaluate the effects of this application on quality of life, symptom management, and patient‐reported outcomes using valid and reliable assessment tools.

The primary strength of this study lies in its comprehensive description of the application development process and the creation of a structured tool designed to address multiple dimensions of postoperative care across different types of breast surgeries. By tailoring educational content according to surgical procedures, the application provides more individualized postoperative guidance.

However, this study had several limitations. First, the number of users involved in the evaluation process was relatively small, which may limit the generalizability of the findings. Second, no control group was included to compare outcomes between patients using the application and those receiving routine postoperative care verbal instructions from healthcare providers. Third, the involvement of the application developer in the final evaluation process may have introduced potential observer bias. Although all users and clinical experts outside the research team reported satisfactory experiences (100%) with the application, this potential source of bias should still be acknowledged. Following completion of the study, the application has also been implemented beyond our institution in other clinical settings and has received consistently positive feedback from both healthcare professionals and patients. Although formal external validation by an independent organization would further strengthen the evidence, this was beyond the scope of the present study and is planned as part of future multicenter evaluations.

Future studies should focus on long‐term clinical and patient‐reported outcomes, including quality of life, symptom relief, adherence to postoperative recommendations, and complication prevention in all types of breast surgeries. Additionally, integrating further interactive and personalized features may enhance the effectiveness of this application in supporting postoperative breast surgery care.

The current version of the application focuses on surgery‐related postoperative care and does not comprehensively cover complications associated with neoadjuvant chemotherapy. Further systematic updating of educational content as well as expansion of them such as treatment‐related adverse effects is important.

Furthermore, the usability of this application can be evaluated using standardized frameworks such as the mHealth app usability questionnaire (MAUQ), the unified theory of acceptance and use of technology (UTAUT) model, and the technology acceptance model (TAM) [21, 22]. In future development phases, integration with hospital electronic health record (EHR) systems and incorporation of AI‐based personalization may be considered to enhance functionality and improve individualized patient support. Additionally, efforts are underway to develop a Progressive Web App (PWA) version and an English‐language version of the application to improve accessibility for a broader population of users.

Moreover, although .NET MAUI represents a promising development framework, it was not adopted in the current version of this application because priority was given to stability and reliability, given that the framework is still relatively new and continues to evolve.

In conclusion, the development and implementation of this mobile application for postoperative care following various breast surgeries demonstrated potential benefits for both patients and healthcare providers. The application offers a user‐friendly platform that may enhance patient engagement and support adherence to postoperative care protocols. Feedback from both patients and clinicians indicated overall satisfaction with the application, suggesting its potential effectiveness in improving postoperative outcomes and enhancing the overall patient experience.

## Author Contributions

B.E., S.A., A.R., and R.O. conceptualized the study. A.R. developed the application. M.A‐K. and B.E. drafted and revised the manuscript and visualized the findings. M.S., M.T., and S.H. reviewed, tested, and provided feedback on the application. S.H. provided patient data for the study. S.A., R.O., M.T., M.S., and B.E. provided educational content.

## Funding

This study was supported by the Tehran University of Medical Sciences (Grant No. 1400‐2‐356‐52742).

## Disclosure

All authors reviewed and approved the final draft of the manuscript.

## Ethics Statement

The Ethical Committee of the Cancer Institute approved this study, with reference number IR.TUMS.IKHC.REC.1400.330.

## Conflicts of Interest

The authors declare no conflicts of interest.

## Data Availability

Data sharing is not applicable to this article as no new data were created or analyzed in this study.
